# Integrated analysis of time- and concentration-dependent metabolomics unravel metabolic changes in raw beef preserved using bacteriocin XJS01

**DOI:** 10.1016/j.fochx.2025.103303

**Published:** 2025-11-19

**Authors:** Jian-Ping Ying, Hai-Yan Wu, Chao-Min Fu, Wei-Wei Li, Lian-Bing Lin, Jun-Wei Xu, Yu-Hang Jiang, Qi-Lin Zhang

**Affiliations:** a Faculty of Life Science and Technology, Kunming University of Science and Technology, Yunnan, Kunming 650500, China; b Department of Cardiology, the Affiliated Hospital of Kunming University of Science and Technology; the First People's Hospital of Yunnan Province, Yunnan, Kunming 650032, China; c Yunnan Key Laboratory of Biodiversity Information, Kunming Institute of Zoology, Chinese Academy of Sciences (CAS), Yunnan, Kunming 650201, China; d College of Food Science, Southwest University, Chongqing 400715, China

**Keywords:** Raw beef, Bacteriocin, Biopreservation, Metabolomics mechanisms, metabolite markers

## Abstract

This study determined the preservation effects of bacteriocin XJS01 on raw beef during storage at 4 °C. An integrated time- and concentration-dependent metabolomic analysis revealed that XJS01 preserved beef by influencing metabolic pathways of spoilage-induced bacteria. These pathways were primarily associated with meat spoilage, cell structure and stress response. The results also demonstrated that the antibacterial activity of XJS01 contributed to preservation by indirectly suppressing the formation of dipeptides. Additionally, 37 metabolites were significantly correlated with meat-quality traits, which were primarily related to valine, leucine and isoleucine degradation and alpha-linolenic acid metabolism. Meanwhile, 1,2,10-trihydroxydihydro-trans-linalyl oxide7-O-beta-D-glucopyranoside and 1-(beta-D-ribofuranosyl)-1,4-dihydronicotinamide were identified as potential biomarkers for estimating beef storage time under XJS01 protection. These findings provide valuable insights into the potential applications of bacteriocins for enhancing preservation and quality assessment in meat.

## Highlights

• Bacteriocin XJS01 maintained meat-quality feature.
• XJS01 preserved beef by affecting meat spoilage and spoilage bacteria pathway.
• XJS01 contributed to beef preservation by supressing generation of meat dipeptides.
• Thirty-seven metabolites were identified to be correlated to meat-quality traits.
• Two metabolites as potential biomarkers for estimating beef storage time.

## 1 Introduction

Beef products are highly perishable due to their low oxidative stability, high unsaturated fatty acids content, and susceptibility to microbial contamination, such as *Escherichia coli* O157:H7. In general, beef spoilage is attributed to four primary factors, i.e., microbial activity, enzymatic autolysis, lipid oxidation, and protein oxidation (Iulietto et al., 2015). These processes lead to protein degradation and the accumulation of nitrogenous compounds, resulting in the formation of toxic organic amines. In addition, protein degradation generates peptides, dipeptides, and free amino acids, which further promote microbial growth. To minimize quality deterioration and extend shelf life, the meat industry employs various chemical antimicrobial compounds. However, numerous studies have raised concerns about the potential health risks associated with the chemical preservatives and antioxidants (e.g., sodium acetate, sodium benzoate, and potassium sorbate), such as their adverse effects on human health (American Academy of Pediatrics, 2018; Hossein, Nader, Karim and Jafar, 2019; Shi Jianrong et al., 2024). Thus, consumer demand for safer and alternative preservation methods for beef products has intensified.

Bacteriocins are colorless, and odorless physical properties, making them an ideal choice for meat preservation and food safety (Xin Weigang et al., 2023; Ying Jianping et al., 2023). Particularly, those produced by lactic acid bacteria (LAB), been proven to be effective in preventing foodborne spoilage (Antonio, Lucas, Hikmate, Eva and Ben, 2008; Ying Jianping et al., 2023). A typical example is that LAB bacteriocins reduce contamination, extending the shelf life of meat products (e.g., the application of plantaricin LFX01 and GZ1–27 in pork preservation (Hechao et al., 2022; Xin Weigang et al., 2023). Bacteriocins can induce damage in cell membrane and genomic DNA as well as alter key metabolic pathways involved in stress response (e.g., two-component system) and membrane transport (e.g., phosphotransferase system) in pathogenic bacteria (Xin Weigang et al., 2023; Ying Jian-Ping et al., 2024). While existing research has focused on the microbiological, biochemical, and sensory aspects of bacteriocins-treated meat, the underlying mechanisms by with bacteriocins contribute to meat preservation remain largely unexplored. Liquid chromatograph mass spectrometer (LC-MS)-based metabolomics has emerged as a powerful tool used to investigate metabolomics related to food characteristics of meats, such as refrigerated goose meat, irradiated marble beef, and Sanhuang chicken (Jingxiu et al., 2023; Zhang et al., 2023; Wen et al., 2025). This provides an opportunity to gain a better understanding of the mechanisms for bacteriocin-based meat preservation.

We previously purified a LAB bacteriocin XJS01 from *Ligilactobacillus salivarius*, demonstrating its broad-spectrum antibacterial activity, high stability, and excellent biosafety profile (Hongwei et al., 2021; Xiang et al., 2022). Moreover, XJS01 exhibited effective preservation effects in raw pork, significantly extending shelf life of meats from 6 to 12 days only at 1-fold minimum inhibitory concentration according to the overall acceptance score in sensory evaluation (Ying Jianping et al., 2023). However, its impact on other economically important meat types, such as beef, remains largely unexplored. In addition, little is known about the relationship between the duration of storage, XJS01 concentration, beef quality parameters, and metabolite profiles.

In this study, the preservation effects of XJS01 on raw beef were investigated based on 12 meat-quality traits, covering color stability, microbiological load, degree of spoilage, and sensory attributes. The primary mechanisms underlying its preservation effects were also explored. In addition, potential metabolites related to meat-quality indexes and metabolite-based biomarkers used for estimating beef storage time were systematically identified. The study uncovered mechanisms related to meat preservation of bacteriocins and provided potential metabolite indicators for bacteriocin application.

## 2 Materials and methods

### 2.1 Preparation of bacteriocin XJS01, bacterial strains, and culture conditions

Bacteriocin XJS01 was purified according to previously established methods (Hongwei et al., 2021; Xiang Yi-Zhou et al., 2022; Ying Jianping et al., 2023). The XJS01 crude extract, derived from L. *salivarius* CGMCC20700, was purified on ÄKTA purifier platform (GE Healthcare, Marlborough, USA) with a Superdex™ 30 column. The resulting purified extract was then freeze-dried using an FD-2 lyophilizer (Bilong, China). XJS01 preparations at a concentration of 1.0 mg/mL were prepared by dissolving XJS01 powder in a 0.1 M phosphate-buffered saline (PBS), and then subsequently utilized in the following experiments.

To accelerate experimental progress and establish a bacterial indicator for assessing the antibacterial activity of XJS01 in beef models, a prevalent beef foodborne pathogen, *E. coli* O157:H7 (henceforth referred to as *E. coli*_O7), was selected for inoculation onto the surface of beef samples. *E. coli*_O7 strain 10,907 was obtained from the China Center of Industrial Culture Collection (CICC, Beijing, China). The strain was resuscitated at 37 °C on nutrient agar and subsequently cultured at 37 °C for 10 h under shaking until reaching the logarithmic phase (approximately 10^7^ CFU/mL), after which it was used for subsequent experiments. The antibacterial activity of XJS01 against *E. coli*_O7 in beef models was determined as previously described (Ying Jianping et al., 2023). Subsequent analysis focused on metabolomics mechanisms and key metabolites involved in beef preservation of XJS01. Despite spoilage is a multi-microbial process, only one bacterial strain was employed as an indicator organism in this study to avoid disturbing influence resulted by a multi-microbial system.

### 2.2 Sample preparation

Raw beef loins (*n* = 7), slaughtered within 12 h, were purchased from seven different food markets in Kunming, Yunnan Province, China. Visible fat and connective tissue were removed, and the meat was cut into small squares (5 × 5 × 2 cm; length × width × thickness) using sterile cutting boards and knives, each weighing approximately 50 g/piece. Meat samples were air-dried at 25 °C until visibly dry.

Subsequently, samples were subjected to dip treatments in sterile distilled water (control group, Con), XJS01 at 1× MIC (low concentration, LC), and 2× MIC (high concentration, HC) for 15 min. The selection of the experimental concentration was performed following the previously described method (Xin Weigang et al., 2023; Deyin et al., 2025), with minor modifications. After treatment, the beef pieces were air-dried at room temperature.

Specifically, *E. coli*_O7 cells were collected via centrifugation at 8000 ×*g* for 5 min. In order to prevent potential interference of excessive *E. coli*_O7 with subsequent metabolomics analyses of beef samples, a low inoculation concentration was employed. A 100 μL aliquot of the bacterial suspension was evenly distributed onto the surface of each beef piece, resulting in a final bacterial concentration of approximately 10^2^ CFU/g. Each sample was independently tested in triplicate.

The selection of the experimental time was performed following the previously described method (Hechao et al., 2022; Deyin et al., 2025), with minor modifications. Beef loins were individually placed on sterile Petri dishes and stored at 4 °C for 12 days. Samples were collected at 0, 3, 6, 9, and 12 post-exposure days (ped) for biochemical, microbiological and sensory analyses. A total of 45 beef loin pieces were used as food models (three biological replicates × five different time points × three distinct XJS01 concentrations).

### 2.3 Determination of meat-quality traits

Microbial growth was assessed based on aerobic mesophilic count (AMC) and *E. coil*_O7 count in beef samples, as previously described (Ying Jianping et al., 2023). In brief, 10 ± 0.1 g of beef was homogenized with 90 mL of saline using a stomacher blender (Stomacher 400 Circulator, UK). The serial 10-times dilutions of the homogenate were carried out with sterile saline. The AMC was determined in Plate Count Agar after incubation at 37 °C for 48 h. *E. coli*_O7 colony showed a blue-green color after 48 h of incubation at 37 °C on sorbitol-McConkey agar (Guangdong Huankai Microbiology Co., Ltd., China) supplemented with 2.5 mg/L potassium tellurite. The tests were performed in triplicate.

For pH measurement, beef loins samples (10 ± 0.1 g) were homogenized in 90 mL of distilled water at 1300 rpm for 10 s using a homogenizer (JRJ300-SH, Yanhe Instrument Equipment Co., LTD, Shanghai, China). The pH was measured using a micro pH-meter model 2001 (Crison Instruments, Spain) with temperature compensation. All experiments were carried out in triplicate.

Total volatile basic nitrogen (TVB-N) values were measured using the semi-micro steam distillation method following the Chinese standard GB 2707–2016. Briefly, 5 ± 0.1 g of beef with 50 mL of deionized water using a homogenizer, transferred all of them to a stoppered Erlenmeyer flask, macerated for 30 min and shook the beaker every 5 min. After filtration, 5 mL of filtrate plus 5 mL of 10 g/L Magnesia were added into a Kjeldahl distillation unit (Anhui Weisi Experimental Equipment Co., LTD, China). During distillation, volatile alkaline nitrogen gas flowed into 10 mL of 20 g/L boric acid solution in a receiving flask along with 5 drops of color indicator. After distillating for 5 min, the boric acid absorbent was titrated using a standard titrant of 0.01 M hydrochloric acid. Ten mL of distilled water was used as a control. TVB-N content was calculated using the equation described in previous studies (Hao et al., 2021; Ying et al., 2023).

Thiobarbituric acid reactive substance (TBARS) values were measured according to previously established protocols (Changbao, Jie, Zhu Xiaoyu, Yingjian and Zhaoxin, 2017; Ying et al., 2023). In brief, 5.0 ± 0.1 g of beef homogenates in 15 mL of 7.5 % trichloroacetic acid solution using a homogenizer. Subsequently, the filtrate (5 mL) was mixed with the 5 mL of 0.02 M 2-thiobarbituric acid reagent, boiled at 95 °C for 30 min and measured at 532 nm using a microplate reader (Thermo Scientific, USA). The blank control was made with 5 mL of distilled water instead of the filtrate. The TBARS values were expressed in terms of mg of malondialdehyde per kilogram of sample (mg malondialdehyde (MDA)/kg).

Meat surface color was measured using a chromatic meter (CR-400, Konica Minolta Investment CO., LTD, China) as described previously (Ying et al., 2023). The colorimeter was calibrated with a white reference board (L* = 95.02, a* = −0.47, and b* = −0.17) before use. Each sample was measured and recorded four times at each sampling point.

Sensory evaluation was conducted based on odor, color, appearance, and overall acceptability, as previously described (Ying et al., 2023). Thirty evaluators aged 20 to 48 years old, non-smokers, and regular consumers of beef products were selected from graduate students and administrative staff at Kunming University of Science and Technology. All participants underwent pre-experimental training (identifying a 1–9 scale [9 = extremely liked, 1 = extremely disliked], reference sample perception exercises), and the scoring scale was calibrated using standard reference samples. During formal evaluation, all randomly coded experimental samples were presented in isolated compartments. Judges conducted five rounds of assessments on days 0, 3, 6, 9, and 12 of the experiment, performing three distinct tests on each sub-sample per round. Judges scored each indicator on a 9-point scale.

### 2.4 Metabolite extraction and ultra-high performance liquid chromatography tandem mass spectrometry (UHPLC) analysis

Based on the observed quality assessment results, the groups showing the most significant differences (3, 6, and 12 ped across all three XJS01 concentrations) were selected for metabolomic analysis. The samples were prepared by mixing the above mentioned three biological replicates, which were further divided equally as seven technical replicates per group/time point. These included three XJS01 concentration groups (control, low concentration, and high concentration) and three time points (3, 6, and 12 days), yielding 63 samples labelled as follows: Con_3, Con_6, and Con_12; LC_3, LC_6, and LC_12; HC_3, HC_6, and HC_12.

Refrigerated beef samples (50 mg) were collected into 2-mL Eppendorf microcentrifuge tubes containing a 6-mm diameter grinding bead. A 400-μL aliquot of extract solution was added, and the samples were ground at 50 Hz for 6 min at −10 °C, followed by ultrasonic extraction for 30 min at 40 KHz. After incubation for 30 min at −20 °C, the samples were centrifuged at 13,000 ×*g* for 15 min at 4 °C. The resulting supernatant was transferred to a fresh glass vial for analysis using an ultra-high-performance liquid chromatography-tandem mass spectrometry (UHPLC-MS/MS) platform. Quality control (QC) samples were prepared by pooling 20-μL aliquots of the supernatant from all samples and mixed them thoroughly.

The analysis of beef metabolites was conducted using a UHPLC-Q Exactive HF-X system (Thermo Scientific, USA) equipped with an ACQUITY UPLC HSS T3 column (100 mm × 2.1 mm i.d., 1.8 μm; Waters, Milford, USA). The mobile phase A consisted of 95 % water with 5 % acetonitrile (containing 0.1 % formic acid), while mobile phase B was composed of 47.5 % acetonitrile, 47.5 % isopropanol, and 5 % water (containing 0.1 % formic acid). The injection volume was 3 μL, and the column was maintained at 40 °C throughout the analysis. The QE HFX mass spectrometer was used for its ability to acquire MS/MS spectra in information-dependent acquisition mode using the accompanying software (Thermo Scientific, USA), which continuously monitored the full-scan mass spectrometry (MS) spectrum. The electrospray ionization source conditions were configured as follows: sheath gas flow rate, 50 Arb; auxiliary gas flow rate, 13 Arb; capillary temperature, 325 °C; full MS resolution, 60,000; tandem MS/MS resolution, 7500; collision energy, 20/40/60 eV in normalized collision energy mode; spray voltage, 3.5 kV or − 3.5 kV.

### 2.5 Identification and annotation of metabolites

Raw data from UHPLC-MS/MS analysis were performed using Progenesis QI software (Waters, USA) for baseline correction, peak identification, integration, retention time correction, peak alignment, and normalization. This comprehensive processing resulted in a data matrix containing critical information, including retention times, mass-to-charge ratios, and peak intensities. The Progenesis QI software was subsequently used to conduct library searches for peak identification. Principal component analysis (PCA) of metabolites can reflect the variation between groups and within groups in general. At the same time, it can not only reduce the dimension and speed up the operation, but also summarize the original data information. Therefore, PCA method is employed to observe the overall distribution trend of inter-group samples and the different degree of inter-group samples in this study. PCA analysis of positive and negative ion was seperately performed to ensure accuracy by cross-validating the results obtained from different ion mode. To perform functional annotation of metabolites, secondary mass spectral matching scores from MS and MS/MS spectral data were used. Metabolites were then searched in the Human Metabolome Database (HMDB) (http://www.hmdb.ca/, accessed on July 2023), METLIN (https://metlin.scripps.edu/, accessed on March 2024), KEGG (Kyoto Encyclopedia of Genes and Genomes, http://www.genome.jp/kegg/, accessed in July 2024), and an in-house MS/MS database (Majorbio Biomedical Technology Co., Ltd. Shanghai, China, https://www.majorbio.com/). The MS mass error threshold was set at <10 ppm for accurate metabolite identification.

### 2.6 Identification of differentially abundant metabolites (DAMs)

DAMs were identified based on the following criteria: variable importance for projection (VIP) > 1; fold change (FC) > 1.2 or < 0.83; and false discovery rate (*FDR*) < 0.05. Metabolite abundance data were tested for normality using the Shapiro-Wilk test. As the data did not conform to a normal distribution, *P*-values were determined using the unpaired Wilcoxon rank-sum test, further corrected using the Benjamini-Hochberg method to obtain *FDR* values. The VIP values were extracted from the performance evaluation results of the orthogonal partial least squares discriminant analysis (OPLS-DA) model using stratified 7-fold cross-validation (k = 7), which included permutation plots and were generated using MetaboAnalystR 4.0 (http://www.metaboanalyst.ca/, accessed in May 2024). The data was log_2_ transformed to mean centering before OPLS-DA. A permutation test (200 permutations) was performed to prevent overfitting (Zhen et al., 2024).

### 2.7 Identification of metabolites significantly related to meat-quality traits by global co-expression network analysis

To identify metabolites closely associated with meat-quality traits, the mean values from all seven samples in each experimental group for all 12 meat-quality traits, which included AMC, *E. coli*_O7 count, pH, TBARS, TVB-N, color changes (L*, a*, and b*), and sensory indicators (color, appearance, odor, and overall acceptability), were analyzed alongside metabolite abundance data across all nine groups. These data were inserted into the weighted gene co-expression network analysis (WGCNA) software in *R* under default parameters (Lise and Orlando, 2023). WGCNA is suitable for studies with >5 experimental groups, with at least three replicates per group in order to generate co-expression modules of metabolites. A similarity matrix of abundance was constructed by clustering metabolotes with correlation. Next, an adjacency matrix converted from the similarity matrix of metabolite abundance was applied to calculate the topological overlap measure (TOM) to determine distance among metabolites, and the topological matrix was further obtained through the adjacency matrix transformation. A clustering dendrogram of the topological matrix was constructed by separating co-expression of abundance modules and merging the modules with similar expression patterns based on TOM dissimilarity. The combination of metabolite abundance and meat-quality values in each module formed metabolite-quality parameter pairs. Metabolite set/modules significantly correlated with meat-quality traits (*P* < 0.05) were extracted for further analysis.

### 2.8 Metabolic pathway enrichment analysis of metabolite sets

KEGG enrichment analysis for targeted metabolite sets was performed using MetaboAnalystR 4.0 with default settings in *R*. *FDR* values were applied to correct *P*-values generated by the software. KEGG pathways with *FDR* < 0.05 were considered significant enriched.

### 2.9 STEM cluster analysis of metabolite abundance change trends

Cluster analysis of DAMs over time was conducted using Short Time-Series Expression Miner (STEM) software (version 1.3.13). The STEM clustering algorithm was applied under default settings, with a significance threshold of 0.05 (Jason and Ziv, 2006). The STEM is suitable for cluster analysis, comparison, and visualization of gene expression data from short time or concentration course (3–8 points) (http://sb.cs.cmu.edu/stem/). In the default mode of trend analysis, the software compared all the following samples with the first one to calculate relative FC values, then normalized using Log_2_ conversion.

### 2.10 Statistics

All data from the three duplications (*n* = 3) were presented as mean ± standard error (S.E.) in assessment for meat-quality traits, and seven duplications were presented (*n* = 7) for change trends of meat dipeptides involved in beef preservation of XJS01, followed with one-way ANOVA plus Bonferroni correction test (threshold of *P*-values = 0.05) in SPSS 22.0 software (IBM Software Inc., NY, USA). The one-way ANOVA were used to assess assumed variance significance among the groups, further analyzed variance targets by the correction test.

## 3 Results and discussion

### 3.1 XJS01 maintained meat-quality traits

**Fig. 1:**
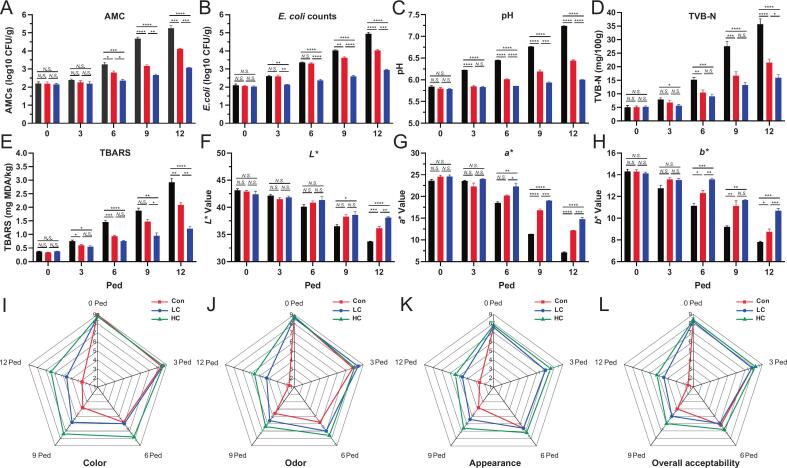
Changes in meat-quality traits during storage. (A) aerobic mesophilic count (AMC), (B) *E. coli* O157:H7 counts, (C) pH, (D) TVB-N values, (E) TBARS values, test color ((F) L*, (G) a*, and (H) b*), and sensory values ((I) color, (J) odor, (K) appearance, and (L) overall acceptability) of raw beef loins without (black column) and with bacteriocin XJS01 protection (low and high concentration represented by red and blue column, respectively). Ped: Post-exposure day. *N.S.*: No significance, *P < 0.05, **P < 0.01, ***P < 0.001, and ****P < 0.0001, the same below. (For interpretation of the references to color in this figure legend, the reader is referred to the web version of this article.)

Here, 12 meat-quality traits were measured. Considering the microbiological parameters, AMC (Fig. 1A) and *E. coli*_O7 counts (Fig. 1B) increased across all three groups over storage time, with the Con group showing the highest increase, followed by LC and HC. At each time point after 6 ped, microbiological count values were significantly (*P* < 0.05) lower in XJS01-treated groups compared to the Con group, particularly in HC. Moreover, a significant reduction (*P* < 0.01) in *E. coli*_O7 counts was detected at 3 ped in HC compared to Con. Finally, microbial populations in LC and HC were approximately 20 % and 40 % lower than of Con, respectively. XJS01 presents antibacterial performance superior to those previously reported, such as *Lactobacillus casei* plus black pepper extract (∼10 % at 12 ped, Shadieh, Javad, Hossein, Leila and Negar, 2022) and phage JN01 (∼15 % at 7 ped, Yaxing et al., 2022). In addition, the antibacterial efficacy of XJS01 on beef is better than that of LFX01 on pork (Xin et al., 2023), nisin on minced sheep meat (Govaris et al., 2010), and plantaricin EmF on beef (Deyin, Wang Qian, Fengxia, Zhao Haizhen and Yingjian, 2022). These findings indicated the strong antibacterial activity of XJS01 in beef preservation, even at low concentrations.

As key indicators for assessing spoilage, fat oxidation, and protein degradation, pH, TVB-N, and TBARS values collectively impacted beef quality (Deyin, Wang Qian, Fengxia, Zhao Haizhen and Yingjian, 2022). Overall, pH values of beef samples increased across all three groups with storage time (Fig. 1C). After 0 ped, at each time point, pH values in LC and HC were consistently and significantly lower than in Con (*P* < 0.001). According to the China National Standard for food safety GB2707–2016, first-class fresh beef must have a TVB-N value below 15 mg/100 g. In Con, TVB-N reached 15.21 ± 0.65 mg/100 g at 6 ped (Fig. 1D), exceeding this threshold. However, LC and HC reached 16.71 ± 1.44 mg/100 g at 9 ped and 15.96 ± 1.12 mg/100 g at 12 ped, respectively. TBARS values showed an increasing trend during storage, with the highest increase in Con, while XJS01-treated groups exhibited significant inhibition (Fig. 1E). In addition, test color values (Fig. 1F-H) declined over time, but the reduction was least pronounced in HC, followed by LC and Con. These findings suggest that XJS01 effectively delays beef spoilage and nutrient loss.

Sensory attributes directly influence consumer acceptance of beef. Sensory scores for color, odor, appearance, and overall acceptability (Fig. 1I-L) showed that the Con group was the first to decrease below the rejection threshold (5 points), followed by LC. Notably, all sensory characteristics in HC remained above 5 throughout the experiment, indicating that XJS01 at high concentrations maintained acceptable beef quality, as similarly observed in raw pork preserved with XJS01 (Ying et al., 2023). These findings demonstrated the ability of XJS01 to preserve meat and extend shelf life, highlighting XJS01 as a potential broad-spectrum preservative for meat products and substitute of chemical preservatives for food safety.

### 3.2 Overview and quality of metabolomics data

**Fig. 2:**
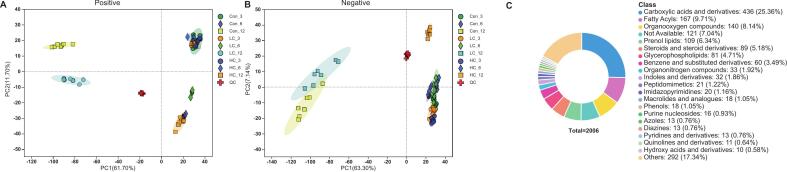
Overview and quality control of metabolomics analysis. Principal Component Analysis (PCA) score plot of all the samples in (A) positive and (B) negative ion mode, respectively; (C) The percentages of detected metabolites in different classes.

A total of 8310 peaks were detected by the mass spectrometer. After quality controls, 7566 peaks were assigned into 2012 metabolites. The correlation values between samples belonging to the same treatment groups in ESI+ (Fig. S1A) and ESI- (Fig. S1B) greater than 0.8. Meanwhile, PCA analysis revealed a consistent pattern across samples (Fig. 2A and B), highlighting robust separation among groups, as further validated by OPLS-DA model analysis (Fig. S2). This confirmed the reliability of the experimental processing and metabolomic sequencing.

**Fig. 3:**
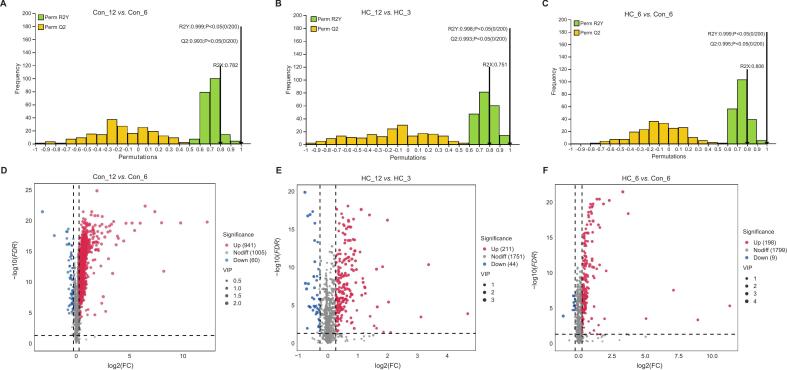
The quality evaluation (A-C) of the OPLS-DA model by three randomly selected comparison pairs and (D—F) the corresponding volcano plot of differential abundance metabolites (DAMs).

A total of 2006 metabolites (Table S1) were detected and classified into 21 categories (Fig. 2C). The reliability of the OPLS-DA models used to calculate VIP values was tested in three randomly selected pairwise comparisons, further ensuring robust identification of DAMs between groups. The models demonstrated excellent predictive performance, with high explained variation (R^2^Y > 0.9) and predictive capability (Q^2^ > 0.5) (Fig. 3A-C), confirming their suitability for further analysis (Zhen et al., 2024). Moreover, volcano plots illustrating DAM distributions (Fig. 3D-F) showed no significant outliers, reinforcing the robustness of DAM screening.

### 3.3 Changes in the metabolic landscape underlying XJS01-mediated meat preservation

**Fig. 4:**
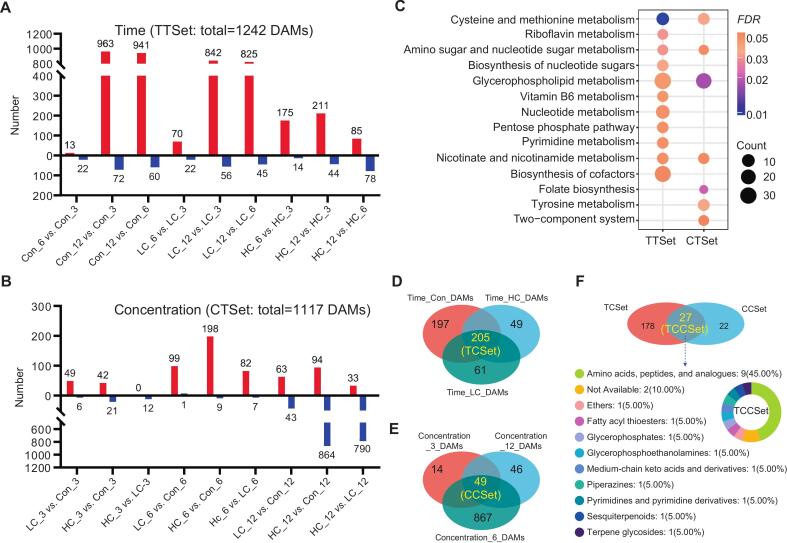
Analysis of metabolites related to beef preservation mediated by XJS01. Number of DAMs of different comparisons in (A) time- and (B) concentration-dependent change; Red and blue columns represented number of up- and down-regulated DAMs, respectively; TTSet: set of total metabolites in time-dependent change; CTSet: set of total metabolites in concentration-dependent change; (C) Integrated bubble chart of KEGG pathways significantly (*FDR* < 0.05) enriched by TTSet and CTSet, respectively; (D) Venn diagram of DAMs identified in Con (Time_Con_DAMs), LC (Time_LC_DAMs), and HC (Time_HC_DAMs) in time-dependent change. TCSet: set of core metabolites in time-dependent change; (E) Venn diagram of DAMs identified at 3 (Concentration_3_DAMs), 6 (Concentration_6_DAMs), and 12 (Concentration_12_DAMs) ped in concentration-dependent change. CCSet: set of core metabolites in concentration-dependent change; (F) Venn diagram of TCSet and CCSet metabolites. TCCSet: set of core metabolites both in time- and concentration-dependent change. The percentages of TCCSet metabolites in different subclasses. (For interpretation of the references to color in this figure legend, the reader is referred to the web version of this article.)

DAMs were identified in 18 comparison pairs, including nine time-dependent (Fig. 4A) and nine concentration-dependent (Fig. 4B) comparisons. A complete list of DAMs for each comparison is provided in Tables S2-S19. In total, 1153 up- and 170 down-regulated DAMs were detected in time-dependent comparisons, whereas concentration-dependent comparisons showed 296 up-regulated and 963 down-regulated DAMs. Therefore, these findings indicate that meat spoilage over storage time induced an overall increase in metabolite abundance, while XJS01 treatment suppresses metabolite levels, particularly at high concentrations in later storage stages (e.g., HC_12 vs. Con_12 and HC_12 vs. LC_12). Similar trends have been observed in beef and chicken treated with antibacterial agents such as oregano essential oil and tannic acid (Marwan, Lee, Aubrey and Ahn, 2016; Yuanpeng et al., 2023), indicating that bacteriocins preserve meat quality by modulating the overall metabolic landscape.

A total of 1242 DAMs were identified as time-dependent metabolites (TTSet), while 1117 were classified as concentration-dependent metabolites (CTSet). Since beef quality declined continuously throughout storage regardless of XJS01 concentration, DAMs in TTSet were considered spoilage-related metabolites, as their abundance changed over time. DAMs in CTSet, which varied according to XJS01 concentration, were identified as metabolites influenced by XJS01 treatment. Enrichment analysis showed that TTSet and CTSet were significantly (*FDR* < 0.05) associated with 11 and 7 pathways, respectively (Fig. 4C). These pathways primarily involved amino acid, nucleotide, and vitamin metabolism, similar to those reported in stored refrigerated beef (Liu Jun, Ziying, Anran and Qin, 2023). Notably, four pathways were identified as spoilage-associated metabolic pathways impacted by XJS01, which included cysteine and methionine metabolism, amino sugar and nucleotide sugar metabolism, glycerophospholipid metabolism, nicotinate and nicotinamide metabolism, as simultaneously enriched in both TTSet and CTSet. These findings suggest that XJS01 helped to preserve beef by affecting nutrition (e.g., animo acids, monosaccharide derivatives, phospholipid, and vitamins)-associated metabolic pathways, thus delaying beef spoilage.

In addition, bacterial stress response and cell structure functional pathways, such as glycerophospholipid metabolism and the two-component system, were also enriched in this study. These pathways have been previously identified as targets of XJS01 in pathogenic bacteria (Tatiana, François, Feuilloley, Nicole and Heipieper, 2015; Ying et al., 2024), suggesting that XJS01 contributes to beef preservation by disrupting stress response and cell structure in spoilage bacteria.

### 3.4 XJS01 inhibited the formation of dipeptides by suppressing bacteria and thus to delay meat spoilage

In this study, 1070, 970, and 342 DAMs were identified in the Con, LC, and HC, respectively, in time-dependent pairwise comparisons. Subsequently, 205 DAMs were classified as time-dependent core metabolites (TCSet) by identifying shared DAMs across all three experimental conditions (Fig. 4D). These were thus considered spoilage-related core metabolites in beef, as they exhibited time-dependent differential responses across all XJS01 treatment groups. Similarly, 49 concentration-dependent core metabolites (CCSet) were identified by intersecting 84, 222, and 1050 DAMs from comparisons at 3, 6, and 13 ped (Fig. 4E). These metabolites were classified as core metabolites influenced by XJS01 exposure, as they exhibited concentration-dependent differential responses at all three time points.

In total, 27 DAMs (TCCSet listed in Table S20) were commonly found in TCSet and CCSet, being identified as core spoilage-associated metabolites affected by XJS01 treatment (Fig. 4F), namely core metabolites involved in XJS01 preservation of beef. Further analysis revealed that dipeptides (marked by red font in Table S20) were overrepresented (15/27, 56 %) in TCCSet, as determined by HMDB classification of metabolites (amino acids, peptides, and analogues accounted for the highest percentage 45 %, Fig. 4F).

**Fig. 5:**
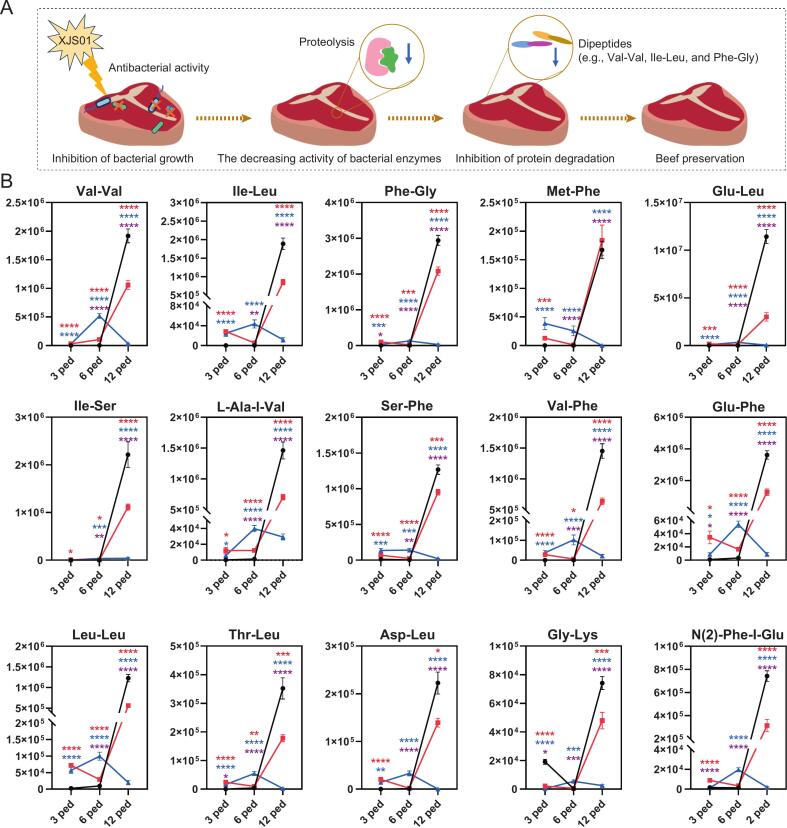
A proposed dipeptide-mediated mechanism underlying beef preservation by bacteriocin XJS01. (A) Summary of the mechanism by which XJS01 inhibits the release of meat dipeptides, thus contributing to beef preservation. (B) Time- and concentration-dependent changes in dipeptide levels during beef preservation mediated by XJS01. Black, and red, and blue lines represented Con, LC, and HC groups, respectively. Red, blue, and purple asterisks represented Con vs. LC, Con vs. HC, and LC vs. HC, respectively. (For interpretation of the references to color in this figure legend, the reader is referred to the web version of this article.)

Dipeptides, including Ile-Leu, Leu-Leu, and Leu-Ile, are small peptides abundant in meat that influence taste and kokumi (kokumi is a taste quality that enhances richness and complexity, thus improving food sensory perception and consumer preference) (Katsuko, Madoka, Akari and Yuji, 2023; Jing, Xuanxiang, Chun and Jun, 2025). Dipeptides are spontaneously produced by the proteolysis implemented either via endogenous enzymes or via the combined effect of endogenous and bacterial enzymes during food processing (Leticia et al., 2015; Fidel, Milagro, Concepción and Leticia, 2018). Given that bacteriocins target bacteria exclusively (Chikindas et al., 2018), we speculated that XJS01 inhibited protein degradation by reducing bacterial enzyme-mediated proteolysis in beef by suppressing growth and/or directly inactivating target bacteria, thereby preventing the formation of dipeptides and thus to delay meat spoilage (summarized in Fig. 5A). This would benefit from further validation, such as enzyme activity assays or microbial community profiling.To further determin this effect, we analyzed abundace profiles of all core dipeptides involved in XJS01-mediated beef preservation (Fig. 5B). Indeed, levels of these dipeptides were significantly lower (*P* < 0.05) in XJS01-treated groups than in Con at the final time point of the experiment (12 ped). In particular, dipeptide abundance remained stable in HC in a time-dependent pattern throughout the experiment, showing the effective suppression of dipeptide formation by XJS01. Interestingly, dipeptide levels were significantly higher (*P* < 0.05) in XJS01-treated groups compared to Con at 6 ped, particularly in HC. This may be explained by a lag in the antibacterial effects of XJS01 prior to this time point due to insufficient action. Moreover, bacterial strains may have strongly increased their metabolic activity (e.g., elevated ATP production and expression up-regulation of protein metabolism-related genes) in response to high XJS01 exposure, which could be considered a stress response to antimicrobial and environmental pressure (Venero et al., 2024; Yuye, Yiwei, Tong, Guiying and Taicheng, 2024).

### 3.5 Metabolites associated with meat-quality traits

**Fig. 6:**
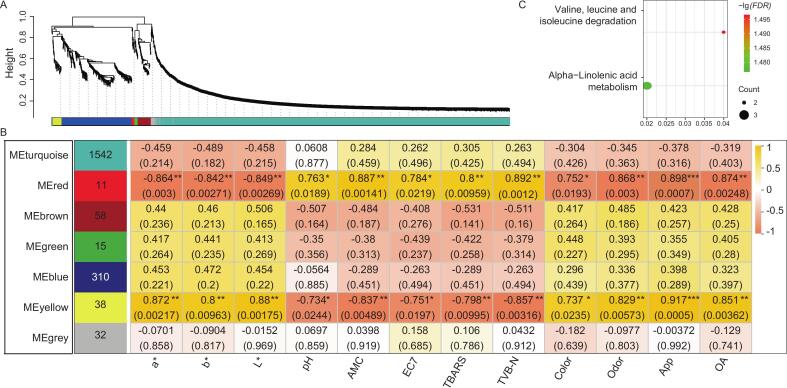
Correlations between metabolites with and twelve meat-quality traits across samples using WGCNA. (A) Clustering dendrogram of the average network adjacency to identify metabolite co-expression modules. (B) Module-trait relationships. (C) Bubble chart of KEGG pathways significantly (*FDR* < 0.05) enriched by MEred plus MEyellow metabolites.

The dendrogram generated by WGCNA clustered identified metabolites into seven modules (Fig. 6A), demonstrating effective module identification. Among these, two modules, MEyellow and MEred, were found to be significantly correlated (*P* < 0.05) with meat-quality traits (Fig. 6B), emphasizing their importance for assessing beef quality. In total, 49 metabolites from these two modules, including 38 MEyellow and 11 MEred metabolites, were significantly enriched (*P* < 0.05) in two pathways, i.e., valine, leucine and isoleucine degradation and alpha-linolenic acid metabolism (Fig. 6C). Previous studies have reported that ω-3 polyunsaturated fatty acids (ω-3 PUFA, e.g., alpha-linolenic acid) and essential branched chain amino acids (e.g., valine, leucine and isoleucine) altered flavor characteristics, shelf-life, and consumer sensory acceptance of beef (Antonelo et al., 2020). A typical example is that the excessive ω-3 PUFA levels have been associated with reduced color shelf life and caused higher fishy and greasy sensory scores. In addition, metabolites involved in valine, leucine, and isoleucine biosynthesis and degradation contribute to beef flavor, tenderness, overall acceptance scores, and muscle quality (Jishan et al., 2025; Antonelo et al., 2020). These findings suggest that the two metabolic pathways identified in this study play key roles in determining beef quality.

**Fig. 7:**
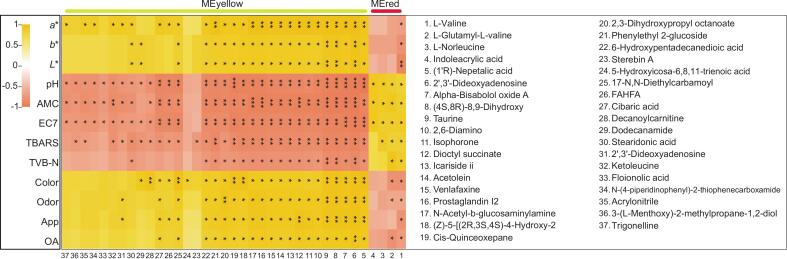
Heatmaps of correlations between meat-quality traits and metabolites that presented significant correlation with at least one trait. EC7: *E. coli* O157:H7 counts, App: appearance, and OA: overall acceptability.

More investigations are required to further understand the exact mechanisms by which various metabolites drive beef quality (Antonelo et al., 2020), we thus further extracted 37 MEyellow and MEred metabolites significantly associated (*P* < 0.05) with at least one quality trait and their correlations with meat-quality traits were mapped (Fig. 7). Four metabolites (No. 1–4) exhibited significantly (*P* < 0.05) negative correlations with meat-quality traits. L-valine was positively correlated with spoilage traits but negatively correlated with quality traits. L-glutamyl-L-valine exhibited a nearly identical correlation pattern, except for test color traits which showed no significance. L-norleucine and indoleacrylic acid were significantly positively correlated (*P* < 0.05) with spoilage traits, particularly pH and microbial counts.

In addition, 18 metabolites (No. 5–22) showed significantly (*P* < 0.05) correlations with all meat-quality traits. Except for TVB-N, it was found that 17-N, N-Diethylcarbamoyl (No. 25) were also significantly (*P* < 0.05) correlated with all other quality traits. Moreover, five metabolites (No. 26–30) were positively correlated with color-related traits of beef but negatively correlated primarily with pH, TBARS, and microbial counts. Seven metabolites (No. 31–37) were predominantly negatively correlated (*P* < 0.05) with pH, TBARS, and microbial counts. Sterebin A and 5-hydroxyicosa-6,8,11-trienoic acid (No. 23–24) were significantly negatively and positively correlated (*P* < 0.05) with TBARS and color, respectively.

To further elucidate metabolite contributions to beef quality, 37 metabolites were extracted based on previously reported metabolic datasets (Table S21), of which 35 (95 %) were derived from meat. Beef-derived metabolites accounted for the largest proportion (21/37, 57 %), followed by those from poultry meat and pork, indicating robust sample handling, experimental progress, and data analysis. Among the meat-derived metabolites, several have been directly associated with beef quality. For instance, taurine is an important nutritional indicator, being highly abundant in beef and essential for human health (Guoyao, 2020). The increasing abundance of valine, caused by an up-regulation of endogenous proteolytic enzyme activity and myofibrillar protein degradation during storage, has been linked to beef color deterioration (Siripong et al., 2025), a finding that aligns with the current study. As an acylcarnitine, decanoylcarnitine has been reported to enhance fat deposition, thereby improving the quality and flavor of yak meat (Pang et al., 2024). While most previous studies have only identified the animal sources of these meat-derived metabolites, their specific roles in meat-quality traits remain largely unexplored. The current study is the first to systematically describe key meat-quality traits associated with these metabolites in beef.

In addition, only one metabolite, N-(4-piperidinophenyl)-2-thiophenecarboxamide, was derived from the dry-aged beef spoilage organism *Pseudomonas lundensis* (Rao Wei, Jinchong, Chen Zhaomin and Jianfeng, 2024). Here, its negative correlation with beef spoilage traits suggested that it may inhibit activity of host spoilage bacteria. This study is also the first to detect alpha-bisabolol oxide A and 2,3-dihydroxypropyl octanoate in meat and to preliminarily find their positive correlation with beef quality. Further validation for these metabolites identified here (especially for novel compounds) is required using physical reference standards (e.g., authenticated compounds) or confirmatory MS/MS spectra in the future.

### 3.6 Potential metabolite indicators for assessing consumed storage time of beef under XJS01 protection

**Fig. 8:**
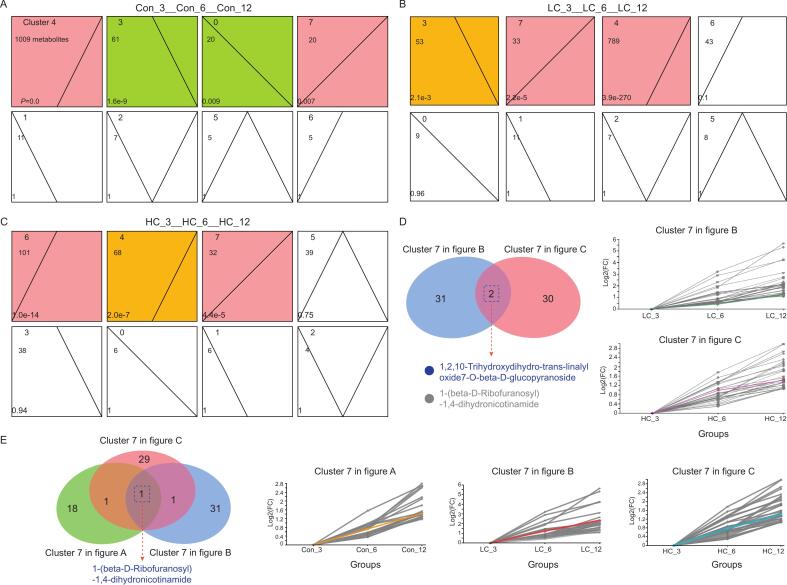
STEM cluster of metabolites with time-dependent changes in (A) Con, (B) LC, and (C) HC. The colored clusters represented significantly (*P* < 0.05) enriched profiles. *P*-values were presented at the bottom left. (D) Venn diagram of metabolites with increasing abundance in time-dependent change in LC and HC (Cluster 7) and the corresponding profiles. Change pattern of 1,2,10-Trihydroxydihydro-trans-linalyloxide7-O-beta-D-glucopyranoside was marked by green and pink lines in LC and HC, respectively. (E) Venn diagram of metabolites with increasing abundance in time-dependent change in Con, LC, and HC (Cluster 7) and the corresponding profiles. Change pattern of DIH was marked by yellow, red, and blue lines in Con, LC, and HC, respectively. (For interpretation of the references to color in this figure legend, the reader is referred to the web version of this article.)

Identification of metabolite indicators for assessing consumed storage time is crucial for measuring beef freshness. Here, four, three, and three significantly clustered sets of metabolites (*P* < 0.05) in time-dependent change trends were obtained using STEM analysis for Con, LC, and HC groups, respectively (Fig. 8A-C). Among these, cluster 7, which included metabolites exhibiting continuously increasing abundance over time, was shared by all three time-dependent comparison groups.

Two metabolites, 1,2,10-trihydroxydihydro-trans-linalyl oxide 7-O-beta-D-glucopyranoside and 1-(beta-D-ribofuranosyl)-1,4-dihydronicotinamide (DIH), were identified as common to both LC and HC groups (Fig. 8D). These metabolites have previously been reported in the metabolite profiles of water-boiled salted duck meat and pig gluteus muscles, where they are associated with meat-quality traits influenced by food processing and breed (Gao et al., 2024; Cong, Sam, Zhou Hui and Baocai., 2022). Their abundance increased in a time-dependent manner in LC and HC groups (Fig. 8D), suggesting that a standard curve could be established to evaluate consumed storage time of XJS01-treated beef based on their levels. In particular, DIH was found in all three continuously increasing clusters (cluster 7, Fig. 8E) across Con, LC, and HC groups, indicating that its accumulation occurred independently of XJS01 exposure and concentration. Changes in time-dependent abundance profiles of metabolites in cluster 7 was constructed for each experimental group (Fig. 8E). Notably, DIH exhibited a nearly uniform increase in relative abundance between consecutive time points, log_2_(FC) values of approximately 0.6, 1, and 0.6 per two-fold time increment in Con, LC, and HC, respectively. This pattern indicates a consistent accumulation of DIH with beef storage time. Thus, DIH may serve as a reliable indicator for accurately assessing the consumed storage time of beef under different conditions.

For application of these potential metabolite indicators in real-world beef monitoring, their abundance need to be accurately measured in target beef samples. Next, determination for a consistent accumulation of levels of metabolits with beef storage time is required, so that we can establish a standard curve used to evaluate consumed storage time of beef based on linear variation in abundance of metabolites. Notably, robustness of the metabolites remains to be validated across breeds, storage conditions, and packaging systems, as found variations of metabolites under various conditions (Bin and Xue-Jun, 2019;Keitiretse and Mulunda, 2019).

## 4 Conclusion

This study determined positive effects of bacteriocin XJS01 on preservation of beef. Moreover, integrated analysis of metabolomics in time and concentration dimensions uncovered primary mechanisms underlying beef preservation of XJS01. Meanwhile, potential biomarkers used for assessment on quality change and consumed storage time were systematically identified for beef. The study uncovered mechanisms related to meat preservation of bacteriocins and provided potential metabolite markers for bacteriocin application.

## Ethics declarations

The Human Ethics Committee of Kunming University of Science and Technology does not have a practice of approving sensory experiments on food. Therefore, this experiment was performed in accordance with the 1964 Declaration of Helsinki and its later amendments and informed consent was obtained from all assessors.

## CRediT authorship contribution statement

**Jian-Ping Ying:** Writing – review & editing, Methodology, Investigation, Data curation. **Hai-Yan Wu:** Writing – original draft, Software, Investigation, Data curation. **Chao-Min Fu:** Writing – review & editing, Methodology, Investigation. **Wei-Wei Li:** Writing – original draft, Methodology, Conceptualization. **Lian-Bing Lin:** Writing – review & editing, Methodology. **Jun-Wei Xu:** Methodology, Investigation. **Yu-Hang Jiang:** Writing – original draft, Methodology, Conceptualization. **Qi-Lin Zhang:** Writing – original draft, Software, Resources, Methodology, Conceptualization.

## Declaration of competing interest

The authors declare that they have no known competing financial interests or personal relationships that could have appeared to influence the work reported in this paper.

## Data Availability

Metabolomic raw data has been submitted to Genome Sequence Archive (accession numbers: OMIX005079).
