# Supplementary material for: Integrated analysis of time- and concentration-dependent metabolomics unravel metabolic changes in raw beef preserved using bacteriocin XJS01
Source: Food Chem X. 2025 Nov 19;32:103303. doi: 10.1016/j.fochx.2025.103303 (PMC12682151; doi:10.1016/j.fochx.2025.103303)
Supplement: Supplementary material 1 — Supplementary materials. [file mmc1.pdf]

Supplementary materials

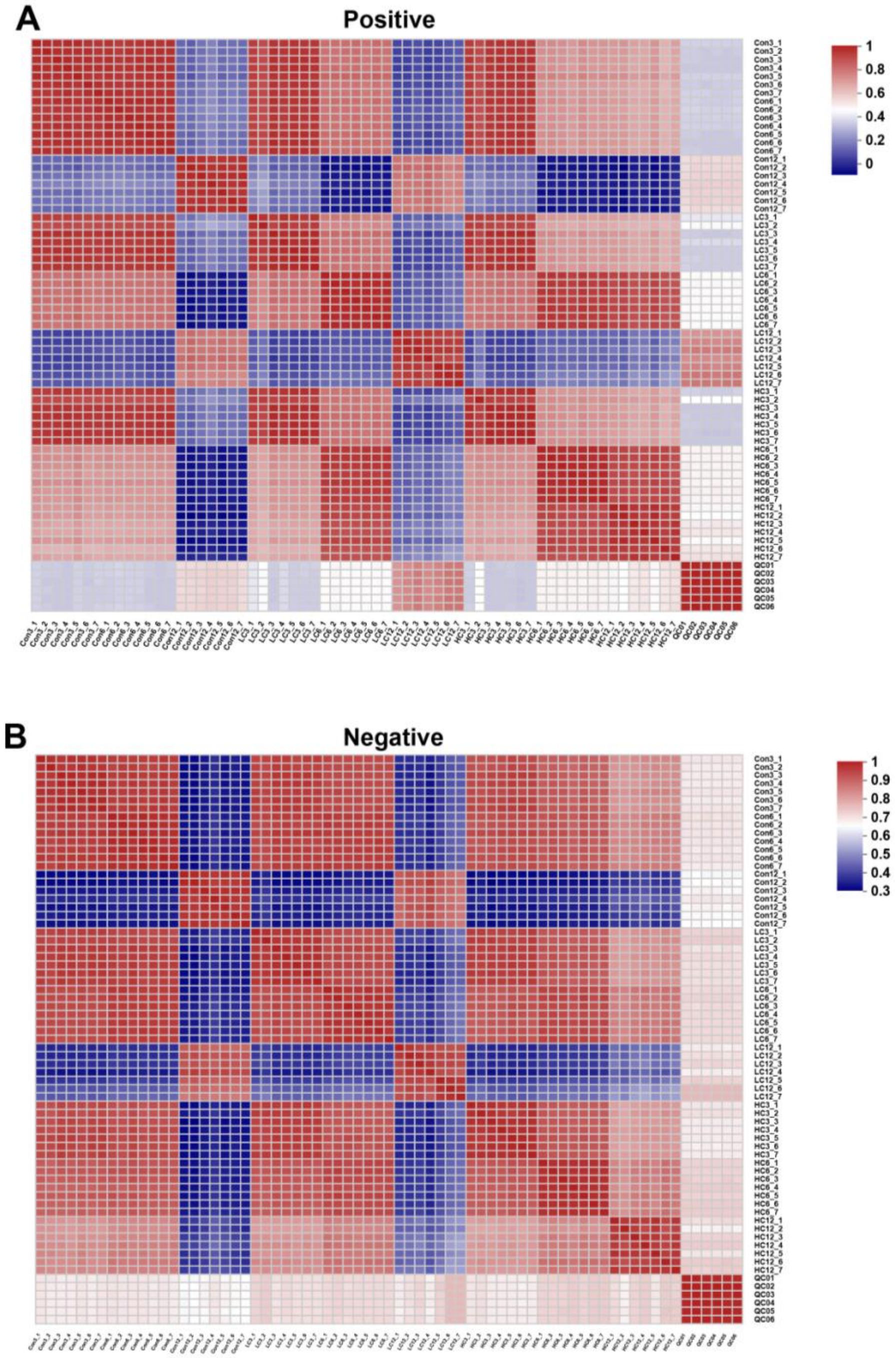

Fig. S1. Heat map of correlation between groups of samples.

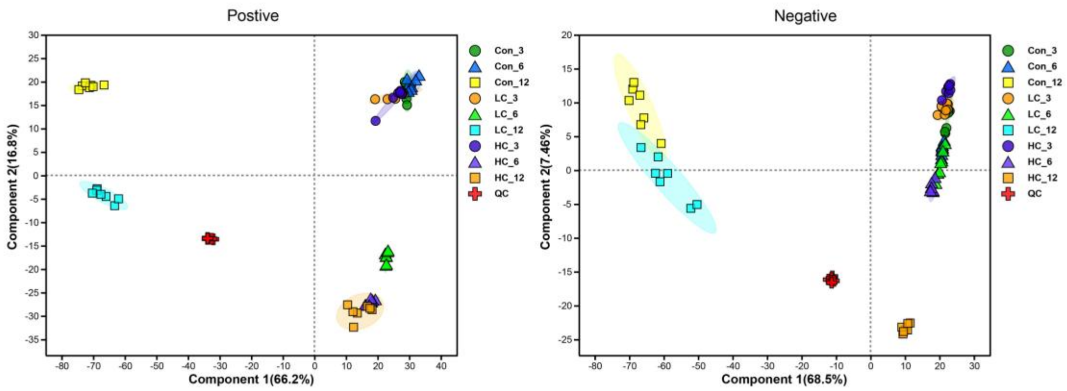

Fig. S2. OPLS-DA cluster of all samples.

Table S21 Information of 37 MEred (1-4) and MEyellow (5-37) metabolites.

No.	Metabolites	Source	Function	References
1	L-Valine	Cattle	Negative correlation with cattle growth	Li et al., 2022
2	L-Glutamyl-L-valine	Beef	/	Yang & Liu, 2019
3	L-Norleucine	Cattle	Related to muscle fiber type	Tan et al., 2025
4	Indoleacrylic acid	Beef	Enhancement of animal immunity	Adeyemi et al., 2020
5	(1'R)-Nepetalic acid	Beef	/	Xu et al., 2024
6	2',3'-Dideoxyadenosine	Rabbit meat	/	Song et al., 2025
7	Alpha-Bisabolol oxide A	/	/	/
8	(4S,8R)-8,9-Dihydroxy	Chicken	Contributed to meat quality	Zhou et al., 2024
9	Taurine	Beef	Contributed to nutrients	Purchas et al., 2004
10	2,6-Diamino	Beef	Contributed to meat quality	Yang & Liu, 2019
11	Isophorone	Chicken	/	Zhou et al., 2024
12	Dioctyl succinate	Beef	/	Xu et al., 2024
13	Icariside ii	Cattle	Related to muscle fiber type	Tan et al., 2025
14	Acetolein	Beef	/	Xu et al., 2024
15	Venlafaxine	Rabbit meat	/	Song et al., 2025
16	Prostaglandin I2	Beef	/	Xu et al., 2024
17	N-Acetyl-b-glucosaminylamine	Beef	/	Xu et al., 2024
18	(Z)-5-[(2R,3S,4S)-4-Hydroxy-2	Chicken	/	Zhou et al., 2024
19	Cis-Quinceoxepane	Beef	/	Xu et al., 2024
20	2,3-Dihydroxypropyl octanoate	/	/	/
21	Phenylethyl 2-glucoside	Cattle	Related to muscle fiber type	Tan et al., 2025
22	6-Hydroxypentadecanedioic acid	Pork	Involved in lipid oxidation	Rocchetti et al., 2020

23	Sterebin A	Duck	/	Li et al., 2022
24	5-Hydroxyicosa-6,8,11-trienoic acid	Yak meat	/	Fu et al., 2024
25	17-N,N-Diethylcarbamoyl	Beef	/	Xu et al., 2024
26	FAHFA	Chicken	/	Zhou et al., 2024
27	Cibaric acid	Beef	Contributed to lipid metabolism	Chen et al., 2022
28	Decanoylcarnitine	Yak	Contributed to fat deposition	Pang et al., 2024
29	Dodecanamide	Cattle	Related to muscle fiber type	Tan et al., 2025
30	Stearidonic acid	Cattle	Related to muscle fiber type	Tan et al., 2025
31	2',5'-Dideoxyadenosine	Rabbit meat	/	Song et al., 2025
32	Ketoleucine	Pork	/	Yeon Jung et al., 2024
33	Floionolic acid	Pork	Involved in animal health	Madsen et al., 2022
34	N-(4-piperidinophenyl)-2-thiophenecarboxamide	*Pseudomonas lundensis*	Involved in beef spoilage bacteria	Rao et al., 2024
35	Acrylonitrile	Chicken	/	Zhou et al., 2024
36	3-(L-Menthoxy)-2-methylpropane-1,2-diol	Duck	/	Li et al., 2022
37	Trigonelline	Beef	Associated with meat quality	Liu et al., 2022

Reference

Adeyemi James A, Peters Sunday O, De Donato, Marcos Cervantes, Andres Pech Ogunade, Ibukun M. Effects of a blend of *Saccharomyces cerevisiae*-based direct-fed microbial and fermentation products on plasma carbonyl-metabolome and fecal bacterial community of beef steers. 2020, *Journal of Animal Science and Biotechnology*, 11,14.

Chen Hao, Wang Chunjie, Huasai Simujide, Chen Aorigele. Metabolomics reveals the effects of high dietary energy density on the metabolism of transition angus cows. *Animals*, 2022, *12*, 1147.

Fu Zheng-Xu, Li Yun-Cheng, Zhang Cai-Ying, Chen Wei-Jun, Meng Fan-Bing, Liu Da-Yu. Nontargeted metabolomics reveals dynamic changes in the quality of fresh yak meat during ice-temperature preservation. *LWT*, 2024, *206*, 116579.

Li Cong, Al-Dalali Sam, Zhou Hui, Xu Baocai. Influence of curing on the metabolite profile of water-boiled salted duck. *Food Chemistry*, 2022, *397*, 133752.

Li Zemin, Shi Jinping, Lei Yu, Wu Jianping, Zhang Rui, Zhang Xiao, Jia Li, Wang Ying, Ma Yue, He Pengjia, Ma Yannan, Cheng Qiang, Zhang Zhao, Zhang Ke, Lei

Zhangmin.. Castration alters the cecal microbiota and inhibits growth in Holstein cattle. *Journal of Animal Science*, 2022, 100(12),367.

Liu Jun, Hu Ziying, Zheng Anran, Ma Qin, Liu Dunhua. Identification of exudate metabolites associated with quality in beef during refrigeration. *LWT*, 2022, *172*, 114241.

Madsen P. A., Curtasu M. V., Canibe N., Hedemann M. S., Pedersen M. L. M., Lauridsen C. Non-targeted metabolomics of saliva to explore potential biomarkers for gastric ulceration in pigs fed hemp. *Animal*, 2022, *16*, 100477.

Pang K., Wang J., Chai S., Yang Y., Wang X., Liu S., Ding C., Wang S. Ruminal microbiota and muscle metabolome characteristics of Tibetan plateau yaks fed different dietary protein levels. *Frontiers in Microbiology*, 2024, *15*, 1275865.

Purchas R. W., Rutherfurd S. M., Pearce P. D., Vather R., Wilkinson B. H. P. Concentrations in beef and lamb of taurine, carnosine, coenzyme Q10, and creatine. *Meat Science*, 2004, *66*, 629-637.

Rao Wei, Wu Jinchong, Fang Ziying, Chen Zhaomin, Wu Jianfeng, Fang Xiang. Antibacterial mechanism of metabolites of *Lactobacillus plantarum* against *Pseudomonas lundensis* and their application in dry-aged beef preservation. *Food Chemistry*, 2024, *460*, 140463.

Rocchetti Gabriele, Bernardo Letizia, Pateiro Mirian, Barba Francisco J., Munekata Paulo E. S., Trevisan Marco, Lorenzo José M., Lucini Luigi. Impact of a pitanga leaf extract to prevent lipid oxidation processes during shelf life of packaged Pork burgers: An untargeted metabolomic approach. *Foods*, 2020, *9*, 1668.

Song Guohua, Solomon Ahamba Ifeanyi, Zhu Tongyan, Li Zhen, Wang Shuhui, Song Bing, Dong Xianggui, Ren Zhanjun. Spatial metabolomics, LC-MS and RNA-Seq reveal the effect of red and white muscle on rabbit meat flavor. *Meat Science*,2025, *219*, 109671.

Tan Xiaofan, Zhao Ruixue, Chen Jing, Yan Zhiwei, Sui Xin, Li Heling, Li Qiao, Du Xuehai, Liu Yangzhi, Yao Siming, Yang Ying, Irwin David M., Li Bojiang, Zhang Shuyi. Integrative transcriptomic, proteomic and metabolomic analyses yields insights into muscle fiber type in cattle. *Food Chemistry*, 2025, *468*, 142479.

Xu Chenchen, Wang Shouwei, Bai Jing, Chen Xiangning, Shi Yuxuan, Hao Jingyi, Zhao Bing. Dynamic microbial community and metabolic profiling in refrigerated beef: Insights from diverse packaging strategies. *Food Research International*, 2024, *197*, 115170.

Yang Bin, Liu Xue-Jun. Metabolite profile differences among different storage time in beef preserved at low temperature. *Journal of Food Science*, 2019, *84*, 3163-3171.

Yeon Jung Doo, Jung Lee Hyun, Kim Minsu, Min Na Kyeong, Yup Lee Do, Jo Cheorun. Metabolomic changes in culture media with varying passage numbers of pig muscle stem cell culture for cultured meat production. *Food Research International*, 2024, *182*, 114138.

Zhang Min, Sun Lina, Su Rina, Corazzin Mirco, Yang Zhihao, Dou Lu, Hu Guanhua, Zhang Yue, Liu Ting, Guo Yueying, Zhao Lihua, Su Lin, Tian Jianjun, Jin Ye. Widely targeted metabolomic analysis reveals the dynamic changes of metabolites during postmortem chilled aging in Mongolian sheep. *Food Chemistry*,

2024, *431*, 137035.
Zhou Zhen, Cai Danfeng, Zhang Zhaofeng, Cai Bolin, Yang Xin, Kong Shaofen, Wu Ruiquan, Lin Duo, Yuan Rongshuai, Mo Yu, Wu Haotian, Nie Qinghua. Metabolomic, lipidomic and transcriptomic reveal meat quality differences among hybrid, indigenous and commercial broiler. *LWT*, 2024, *209*, 11676.
